# *Arthrobacter* sp. Inoculation Improves Cactus Pear Growth, Quality of Fruits, and Nutraceutical Properties of Cladodes

**DOI:** 10.1007/s00284-023-03368-z

**Published:** 2023-07-03

**Authors:** G. Platamone, S. Procacci, O. Maccioni, I. Borromeo, M. Rossi, Loretta Bacchetta, C. Forni

**Affiliations:** 1 PhD School in Evolutionary Biology and Ecology, University of Rome “Tor Vergata”, Rome, Italy; 2 Department of Biology, University of Rome “Tor Vergata”, Via della Ricerca Scientifica, Rome, Italy; 3 Bioproducts and Bioprocesses Laboratory, BIOAG Division, SSPT Department, ENEA Casaccia, Via Anguillarese 301, Rome, Italy

## Abstract

A study was undertaken to determine the effects of a strain of *Arthrobacter *sp., a Plant Growth-Promoting Bacteria (PGPB), on plant phenology and qualitative composition of *Opuntia ficus-indica* (L.) Mill. fruits and cladodes. The strain was inoculated in soil, and its effects on cactus pear plants were detected and compared to nontreated plants. Compared to the latter, the treatment with bacteria promoted an earlier plant sprouting (2 months before the control) and fruitification, ameliorating fruit quality (i.e., improved fresh and dry weight: + 24% and + 26%, respectively, increased total solid content by 30% and polyphenols concentrations by 22%). The quality and quantity of monosaccharides of cladodes were also increased by *Arthrobacter *sp. with a positive effect on their nutraceutical value. In summer, the mean values of xylose, arabinose, and mannose were significantly higher in treated compared to not treated plants (+ 3.54; + 7.04; + 4.76 mg/kg d.w. respectively). A similar trend was observed in autumn, when the cladodes of inoculated plants had higher contents, i.e., 33% xylose, 65% arabinose, and 40% mannose, respect to the controls. In conclusion, *Arthrobacter *sp. plays a role in the improvement of nutritional and nutraceutical properties of cactus pear plants due to its capabilities to promote plant growth. Therefore, these results open new perspectives in PGPB application in the agro-farming system as alternative strategy to improve cactus pear growth, yield, and cladodes quality, being the latter the main by-product to be utilized for additional industrial uses.

**Supplementary Information:**

The online version contains supplementary material available at 10.1007/s00284-023-03368-z.

## Introduction

The Farm to Fork Strategy is the heart of the European Green Deal aiming to make food systems fair, healthy, and environmentally friendly. Therefore, following EU strategy, the current agriculture is implemented by more sustainable cultivation practices. The use of Plant Growth-Promoting Bacteria (PGPB) in many crop species has proven to be a promising practice in sustainable cultivation due to their positive effect**s** on plant growth, yield quantity and quality, and on better adaptability to both biotic and abiotic stresses [1–3]. These microorganisms**,** applied to the soil can act on the plant either directly by producing (i.e., synthesis of plant growth regulators hormones, volatile organic compounds, trehalose, exopolysaccharides**)** and/or regulating the metabolism of different molecules (e.g., by ACC deaminase activity, or indirectly via pathogen antagonism or induced systemic resistance [1, 4, 5]). Several studies have shown the positive effect of PGPB on different crops [2, 6–9], thus, implementing the interest towards such promising bacterial strains and their possible application in agronomic field. Among plant growth-promoting species, *Arthrobacter *sp. has shown a high adaptability to interact with different plant species [2, 10–13]. The strain was originally isolated from the leaf cavities of the fern *Azolla filiculoides* Lam., where the bacteria are also localized within the sporocarps [12–14]. Further characterization of the bacteria included the synthesis of polysaccharides [14] and IAA [15], halotolerance and lack of ACC deaminase activity [2, 10, 16], and the ability to help plants in counteracting salt stress [2, 10]. *Actinobacteria* have been also isolated from *Opuntia ficus-indica,* the molecular phylogeny of the isolated strains revealed the components of the distinct clades of *Streptomyces* spp. quite away from other actinobacterial genera [17, 18]; these bacteria may play a role in drought-tolerant species with potential amelioration of plant growth, especially under abiotic stress conditions. Basing on the under-exploited plant-bacteria interactions in cactus species, and the promising results obtained in other crops, the aim of this work was to explore the potential compatibility of the use of *Arthrobacter *sp. in drought-tolerant crop. *Opuntia ficus-indica* (L.) Mill. (OFI), which belongs to the family *Cactaceae*, is an important crop in agricultural economies, mainly in arid and semi-arid parts of the world, due to its crassulacean acid metabolism (CAM) and its great efficiency in water utilization and conservation [19, 20]. Its fruits are very popular and appreciated in many countries for their nutraceutical and nutritional properties with an increasing industrial interest on cladodes as raw materials for feed, food, cosmetic, pharmaceutical applications [21–24]. The great amount of mucilage**,** mainly composed by high molecular weight polysaccharides (arabinose, xylose, rhamnose)**,** makes cladodes a remarkable source for hydrocolloid market addressed to functional ingredients which is expected to reach 13.36 billion USD by 2026 [24]. Italy is the most important supplier of cactus pear in Europe with the cultivation mainly located in Sicilian specialized orchards (90% of the national production) [25]. Although this crop is considered a rustic species, the implementation of modern cultivation practices enhances crop productivity, quality, and sustainability; especially in organic agriculture system, the request for plant bio-stimulants, biological fertilizer, or biofertilizer is increasing in the last years. Therefore, this study aims to contribute to the discussion on the effectiveness of using PGPB in agriculture. More specifically, the aim of this work lays on *Arthrobacter *sp. soil inoculation and its effects on cactus pear plant growth, yield, and cladodes quality. To this end, the study of biometric parameters, i.e., cladodes and fruits (weight sizes), as well as biochemical characteristics (polyphenols, antioxidant activity, polysaccharides content) were quantified as measures of the plant fitness and compared with those obtained from not inoculated plants. The use of alginate beads as bacteria inoculum tool has been also considered.

## Experimental Setup

### Bacteria Inocula Preparation

*Arthrobacter *sp. strain CD belongs to the collection of the Laboratory of Botany and Phytotechnologies, Department of Biology, University of Rome Tor Vergata. The strain, isolated from *Azolla* leaf cavities, was identified according to standard procedure and compared with *Arthrobacter* *globiformis* type strain  ATCC 8010 of Pasteur Institute [26].

The strain was stored in glycerol solution at − 80 °C. The bacteria were grown in tryptic soy broth (TSB) medium (Sigma-Aldrich) at 30 °C and refreshed twice a week with fresh medium.

To encapsulate, formulate, and apply *Arthrobacter* as bioinoculant, alginate beads were considered [16, 27]. The bacterial culture (OD_600_ = 1.3) was diluted (1:2 v/v) in a sterile solution of Na^+^-alginate (6% w/v) to a final concentration of 3% alginate. The addition of sterile CaCl_2_ solution (0.2 M) allowed the formation of beads [16]. The beads were washed 3 times with sterile milli-Q water and stored at room temperature in sterile conditions.

The inoculum was prepared by re-suspending the beads in medium as follows. Fifty-six beads were divided into 7 tubes of 50 mL volume. Twenty mL of minimal culture medium (saline solution (0.9% NaCl) added with 2.5 g/L glucose) was added to the tubes. After 48 h, the cultures (OD _600 nm_ = 7 × 10^6^ CFU) were ready to be inoculated near the roots of acclimated plants.

### Plant Materials and Phenological Evaluations

Cladodes were collected in specialized cactus pear orchard of the Gaetano Spitale farm located in Mazzarino (CL) Italy, 37°16′14.9"N 14°20′17.4"E. The cladodes (2 years old) were immediately planted in pots, containing the same soil of the field (sand 724 g/kg; silt 70 g/kg; clay 206 g/kg; pH 7; electrical conductivity 0.7 dS/m; total porosity 80% (v/v). The temperature at the time of transplanting was 23 °C and RH 68%. The plants were acclimated for 5 months, and then inoculated as reported above. A complete randomized block design was applied in the experiments: i.e., the pots were randomly divided in two sets, a first set was inoculated with bacteria, and the other noninoculated was used as control. Each month the growth of the plants was evaluated by (1) time of sprouting, (2) number and size of new cladodes, (3) flowering time, and (4) number of fruits per plant. Phenological observations were conducted for 2 years.

Samples of cladodes (in summer and autumn) and fruits (in autumn) were collected from inoculated and not inoculated plants. All samples were oven dried at 60 °C for 72 h and maintained in dry conditions until analyses.

### Evaluation of Fruit Characteristics

Fresh and dry weights were determined on raw or dried cactus per fruits; total soluble solids (% Brix) were evaluated on juices, obtained from fruits peeled and homogenized by blade homogenizer. The juices filtered and diluted (1:10 v/v) in distilled water were analyzing using HI96801 refractometer (Hanna instrument).

### Extraction and Quantification of Total Phenolic Compounds

Total polyphenols and antioxidant activity were evaluated on dried cladodes and fruits. We used the Folin–Ciocâlteu method as reported by Procacci et al. [22] with slight modifications. Briefly, an aliquot of 200 µL of standard solution, extract of sample (40 mg in 1 mL ethanol 80% at 30 °C for 30 min), or 80% methanol (control) was mixed with 1.5 mL of Folin–Ciocâlteu reagent (Sigma-Aldrich), which was previously diluted with double-distilled water (1:10). The mixture was allowed to stand for 5 min, and then 1.5 mL of sodium carbonate (60 g/L) was added to the mixture. Finally, the mixture was allowed to stand in the dark for about 30 min and the absorbance (Abs) was measured at 725 nm against a blank solution using a UV/Visible spectrophotometer (Lambda 2—Perkin Elmer). All measurements were carried out in triplicate, and results were expressed as milligram of gallic acid eq/g dry weight (mg Gallic Acid eq/g d.w.). A calibration curve was performed with gallic acid as standard (*y* = 0.0031*x − *0.0338, *R*^2^ = 0.9981).

Flavonoids were estimated following this procedure [28]: 200 mg of dry material was homogenized in liquid nitrogen and re-suspended in 5 mL ethanol (95% v/v), incubated overnight at 4 °C, and then centrifuged at 8000 g for 15 min. The supernatants were stored at − 20 °C until analysis. The sample absorbance was measured at 415 nm. To calculate the concentration of flavonoids, a calibration curve was performed using quercetin (Sigma) as standard at different concentrations (10 μg/mL, 20 μg/mL, 40 μg/mL, and 80 μg/mL; *y* = 0.0081*x − *0.0321, *R*^2^ = 0.999). Flavonoids are expressed as μg quercetin eq/g d.w.

### Antioxidant Activity

Antioxidant capacity was determined by DPPH method [22]. Briefly, 40 mg of each sample was extracted with 1 mL of 70% methanol at 30 °C × 30 min and filtered at 0.2 µm. 100 µL of each extract was, thus, added to 3.9 mL of a DPPH radical solution 0.06 mM, mixed in a Vortex and kept in the dark conditions × 60 min before reading the absorbance at 517 nm (spectrophotometer Varian CARY 50 Scan). The antioxidant activity was obtained from the decrease in absorbance of DPPH (% inhibition), from which, by interpolation, the results were expressed as mg of Trolox equivalent per gram of dry weight (µmol TE g/d.w.). The percentage of inhibition was obtained by interpolation of a calibration curve (*y* = 0.1164*x* + 5.0225, *R*^2^ = 0.9999) (Abs vs. µM Trolox)

$$\% \;{\text{inib}}{.} = 1 - \left( {A_0 /A_{\text{s}} } \right) \times 100,$$

*A*_0_ = absorbance of control sample (*t* = 0 h) and *A*_s_ = absorbance of a tested sample at the end of the reaction.

### Cladodes Sugar Composition

The sugars were extracted from 10 g of the finely ground-dried cladode sample with a mixture acetonitrile/water 60:40 (v/v) under constant stirring for 3 h. Monosaccharide’s composition was evaluated by HPLC using specific chromatographic column (Amino Carbohydrate Waters, Millipore); the revelation was carried out by means of a refractive index detector. The HPLC pump has been set up to flow approximately at 1.0 mL/min, and mobile phase was acetonitrile/water 75:25 (v/v). The content of the individual monosaccharides, expressed as a percentage, was calculated according to the following formula:

$${\text{Monosaccharide}}\,\left( {\text{g/100g}} \right) = \left( {A_{\text{c}} /A_{\text{s}} } \right) \times M_{\text{s}} /M_{\text{c}} \times 100,$$

*A*_c_ = area of the sample peak, *A*_S_ = area of the peak of the standard, *M*_s_ = quantity of standard injected expressed in g, and *M*_c_ = quantity of sample injected expressed in g [29].

### Statistical Analysis

The experiments were carried out in three replications. Data of biological feasibility studies were subjected to one-way Analysis of Variance (ANOVA) by program Jamovi software version 2.3.1. Data means were compared with Fisher’s protected Least-Significant Difference (LSD) test at probability level of 0.05. Differences at the *P* ≤ 0.05 levels were considered significant. When comparing inoculated groups with noninoculated ones, the significance was reported as ***P < 0.001; **P < 0.01; *P < 0.05.

## Results

### Effect of Bacteria on Plant Phenological Phases

**Fig. 1:**
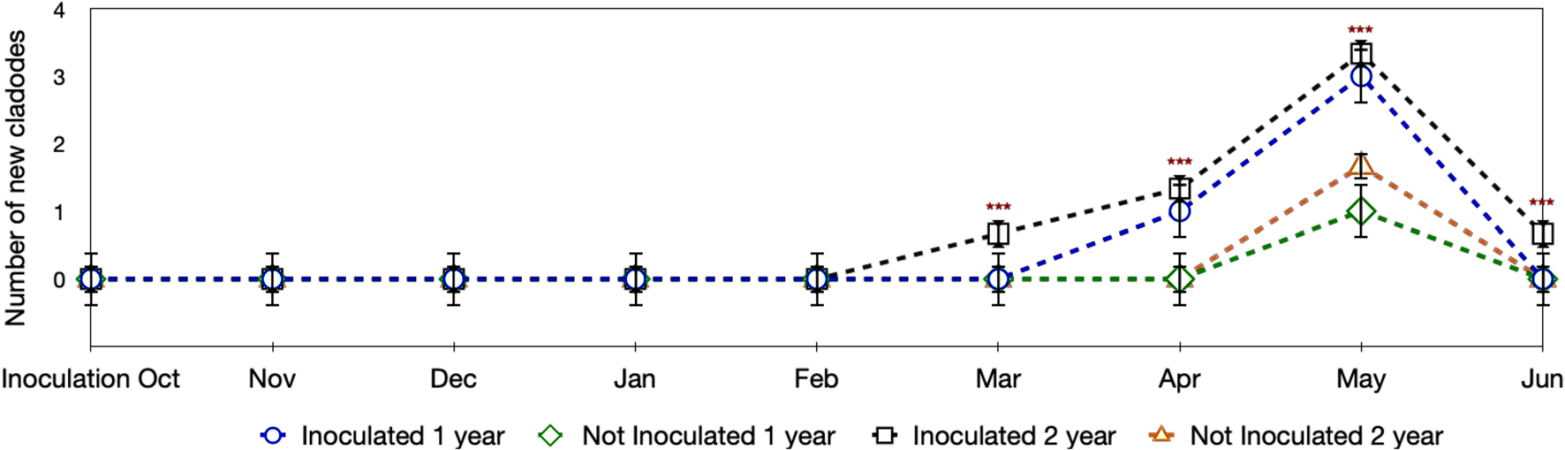
Number of new cladodes developed in plants treated with *Arthrobacter *sp. compared to the control. Inoculated plants anticipated sprouting of 1 or 2 months during the first and the second years of evaluation, respectively. Data collected from October to June in the 2 years of the experiment are expressed as mean ± SD of three replications of five plants each. Significant differences between not inoculated and inoculated plants by ANOVA test determined by ANOVA test, are reported as ***P < 0.001

Inoculated plants started to produce new cladodes 3–4 months after the treatment, a delay of 1 or 2 months in sprouting was observed in the controls both in the first and second years of experiment (Fig. 1). Significant differences were also found both in the number and size of newly differentiated cladodes. As reported in Fig. 1, inoculated plants formed 3–4 cladodes/plant from February–March to May with the untreated plants 1–2 cladodes/plant from April to May (data confirmed in the 2 years of observation). Furthermore, the surface area of cladodes after 1 month from differentiation was greater in treated plants (+ 56.03% ± 1.31 and + 57.53% ± 1.28 in the first and the second years, respectively) when compared to the newly pad average area of the control (see Table A_Online Resource 1).

Therefore, the time of flowering was anticipated of about 2 months in inoculated plants; consequently, the inoculated plants prolonged fruiting season providing a second harvest at the beginning of autumn season (average 6.0 ± 0.5 fruits/plant), when the controls just developed the first fruits (not more than 2–3 for year).

### Effect of Bacteria on Physio-biochemical Parameters of Fruits

The inoculation with *Arthrobacter *sp. positively influenced the fruit weight with an increase equal to 31% in fresh fruit weight, which led to a higher dried weight (+ 26%) and total soluble solids (+ 3.87% °Brix) when compared to untreated plants (Table 1).

**Table 1:** Effects of *Arthrobacter*  sp. on cactus pear fruit quantity and quality

Fruit samples	Fruit number	Fresh weight (g)	Dry weight (g)	Total soluble solids (°Brix)	Total phenolic compounds (mg gallic acid eq./g d.w.)	Total flavonoids (µg quercetin eq./g d.w.)	Antioxidant activity (µmol Trolox eq./g d.w.)
Not inoculated	2.5 ± 0.5	65.1 ± 1.9	13.7 ± 0.5	9.3 ± 0.2	12.2 ± 3.65	5.9 ± 2.3	13.9 ± 5.0
Inoculated	6.0 ± 0.5	85.3 ± 2.9***	17.3 ± 0.2***	13.2 ± 0.4***	15.6 ± 1.8*	7.6 ± 3.7	15.6 ± 0.70

The inoculation with bacteria positively influenced the fruit weight, the total soluble solids, as well as the total polyphenol content and the antioxidant activity. No significant effect was observed for total flavonoids content of in treated or not treated samples. Data are expressed as mean ± SD of three replications/plant collected in the 2 years of experiments. Significant differences, between inoculated and noninoculated plants (control) determined by ANOVA test, are reported as *P < 0.05, ***P < 0.001

Moreover, *Arthrobacter *sp. positively affected the total polyphenol (TP) content of the fruits collected in August. As reported in Table 1, the mean value of TP was significantly higher in treated (15.6 mg Gallic Acid eq/g d.w.) compared to nontreated plants (13.94 mg Gallic Acid eq/g d.w.)

Non significant effect was observed for total flavonoids content of samples (values ranged from 5.94 µg quercetin eq./g d.w. in nontreated plants to 7.65 µg quercetin eq./g d.w. in treated ones).

Data, from DPPH assays on fruits, revealed the positive influence of bacteria inoculation on antioxidant activity. The samples of treated plants showed an increment of bioactive molecules activity if compared to untreated ones (15.6 µmol Trolox eq./g d.w., 10.9 µmol Trolox eq./g d.w., respectively).

### Effect of Bacteria on OFI Cladodes Nutraceutical Composition

**Fig. 2:**
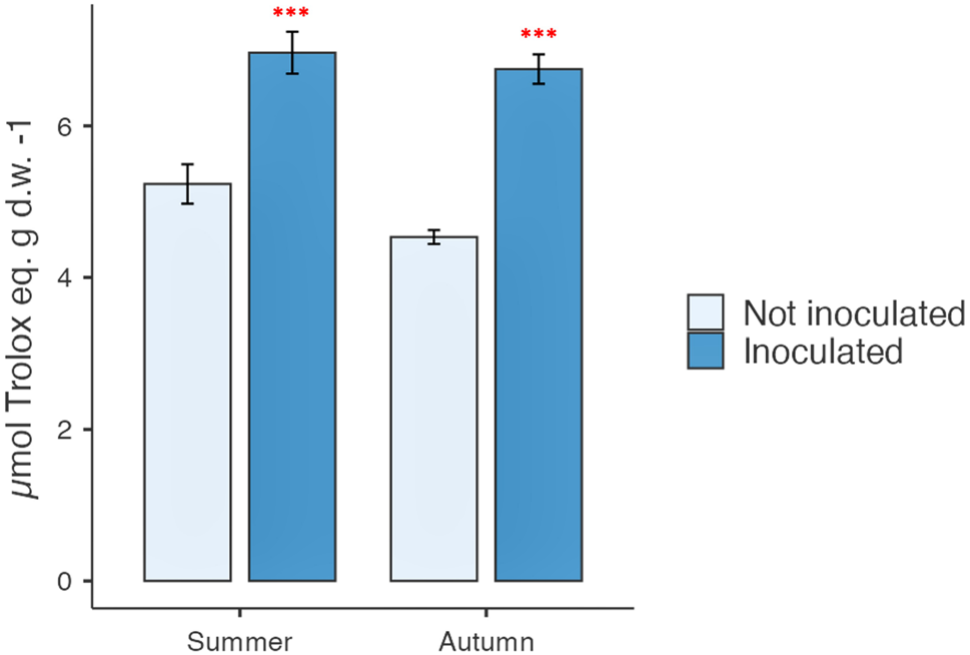
Effect of *Arthrobacter *sp. inoculation on monosaccharide composition of cladodes sampled in summer and autumn (a = Xylose; b = Mannose; c = Arabinose). The presence of bacteria significantly enhanced the amounts of xylose, mannose and arabinose in cladodes when compared to those of noninoculated plants both in summer and autumn. Data, determined by HPLC analysis in triplicate, are expressed as mean ± SD. Significant differences between noninoculated and inoculated plants determined by ANOVA test are reported as ***P < 0.001

The presence of bacteria significantly enhanced the amounts of xylose, mannose, and arabinose in cladodes (Fig. 2). In summer, the mean values of these monosaccharides were significantly higher in treated compared to untreated plants (29.11 mg xylose/kg d.w. vs. 25.57 mg xylose/kg d.w.; 31.01 mg arabinose/kg d.w. vs. 24.01 mg arabinose/kg d.w. and 22.20 mg mannose/kg d.w. vs. 17.44 mg mannose/kg d.w.). A similar trend was observed in autumn (40.86 mg xylose/kg d.w. vs. 60.93 mg xylose/kg d.w.; 1.06 mg arabinose/kg d.w. vs. 3.06 mg arabinose/kg d.w. and 22.60 mg mannose/kg d.w. vs. 13.46 mg mannose/kg d.w.)

**Fig. 3:**
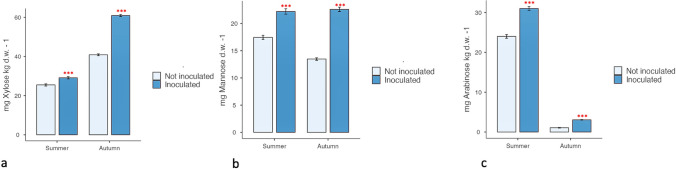
Total phenolic content in cladodes sampled in summer and autumn from plants inoculated and noninoculated with *Arthrobacter *sp. Results of the Folin–Ciocâlteu assay, indicated a minor content of total polyphenols in plants inoculated with bacteria; these differences were significantly marked in cladodes collected in June respect to those sampled in autumn. Data are expressed as mean ± SD of three replications and three spectrophotometric readings each one. Significant differences by ANOVA test are reported as ***P < 0.001 and *P < 0.05

Results of the Folin–Ciocâlteu assay (Fig. 3) performed on cladode samples, indicated a minor content of total polyphenols in plants inoculated with *Arthrobacter *sp. These differences were significantly marked in cladodes collected in June (14.5 mg Gallic Acid eq/g d.w. in inoculated vs. 23.54 mg Gallic Acid eq/g d.w. in noninoculated) respect to those sampled in autumn (17.23 mg Gallic Acid eq/g d.w. in inoculated and 18.25 mg Gallic Acid eq/g d.w. in noninoculated).

**Fig. 4:**
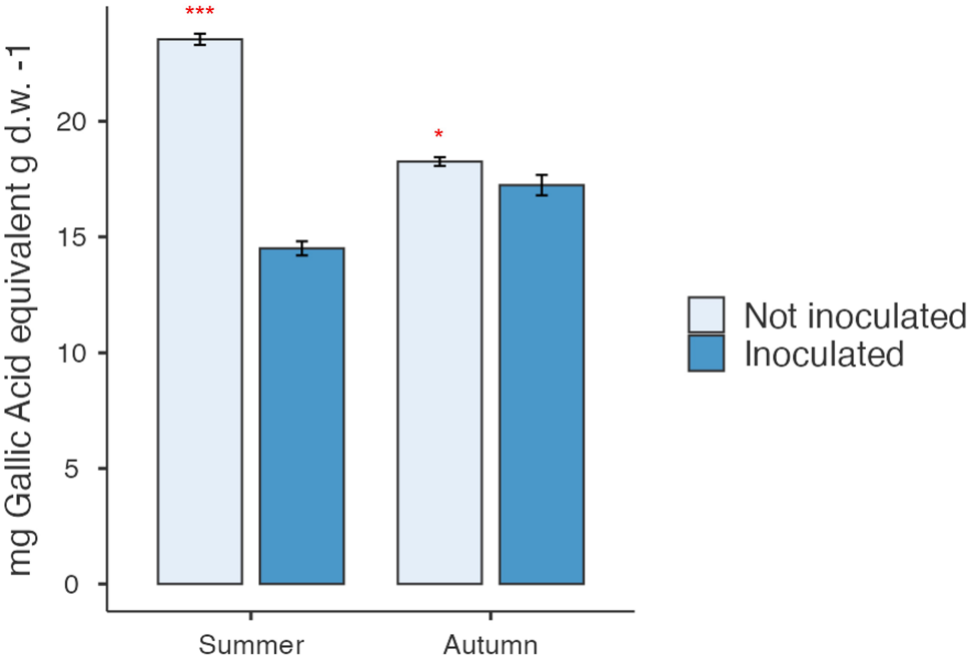
Flavonoids content in cladodes sampled in summer and autumn from plants inoculated and noninoculated with *Arthrobacter *sp. The content of flavonoids was significantly lower in samples from plants treated with bacteria respect to those untreated. No differences were found in the same samples collected in autumn, which were characterized by a minor content of flavonoids if compared to those of summer season. Data are expressed as mean ± SD of three replications and three spectrophotometric readings each one. Significant differences by ANOVA test are reported as **P < 0.01

Among the total polyphenols, the content of flavonoids, which are a characteristic component of cactus pear, was significantly lower in samples from plants treated with bacteria (51.54 µg quercetin eq/g d.w.) respect to those untreated (73.87 µg quercetin eq/g d.w.) (Fig. 4). No differences were found in the same samples collected in autumn, which were characterized by a minor content of flavonoids (37.79 µg quercetin eq/g d.w. and 38.10 µg quercetin eq/g d.w. respectively) if compared to those of summer season.

**Fig. 5:**
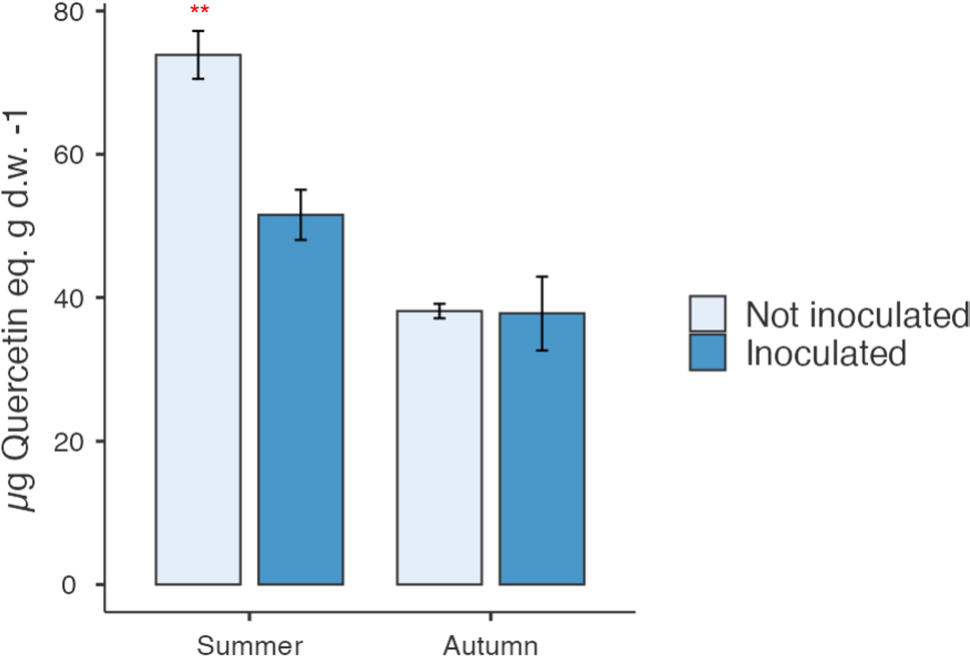
Antioxidant activity in cladodes sampled in summer and autumn from plants inoculated and not inoculated with *Arthrobacter *sp. The antioxidant activity performed by DPPH method showed that the antioxidant activity in cladodes was increased in inoculated samples, collected in both summer and autumn when compared to the controls. Data are expressed as mean ± SD of three replications and three spectrophotometric readings each one. Significant differences by ANOVA test are reported as ***P < 0.001

The antioxidant activity performed by DPPH method is reported in Fig. 5. Data showed that the antioxidant activity in cladodes was increased in inoculated samples, collected in both summer (6.96 µmol Trolox eq/g d.w.) and autumn (6.75 µmol Trolox eq/g d.w.) when compared to the controls (5.23 µmol Trolox eq/g d.w. and 4.53 µmol Trolox eq/g d.w.), respectively.

## Discussion

Under the perspective of climate changes, increasing of soil salinization and aridity, the possibility of using PGPB can represent a useful approach to improve plant tolerance to abiotic stresses. Among these bacteria, *Arthrobacter *sp. has been proved to be an excellent microbial inoculum in rapeseed exposed to salinity [2, 10, 11]. Besides of living in the symbiotic association of *Azolla-Anabaena* [15], this species is also widespread in soils [30, 31]. Moreover, the possibility to use bioencapsulated microorganisms as inoculants represents a further benefit to a wider utilization of bacteria inocula [27].

Since *Arthrobacter *sp. can produce IAA [15], thus, influencing cactus pear growth anticipated the yield, and improved some qualitative characteristics of fruits and cladodes. In fact, inoculated plants differentiated more cladodes of bigger sizes, and anticipated the flowering (1–2 months before the control), with an enhancement of the number of fruits per plants (see Figure A_Online Resource 2). Based on these results, the opportunity to lengthen the fruit picking period provides positive impact on the fruit supply and commercialization, which is particularly interesting for the national and international markets. In similar research, Morais et al. [7] recently reported that PGPB inoculation significantly accelerated crop maturation in strawberry. The same authors proved also the inoculation with a strain of *Pedobacter*, led to an enhancement in the dimensions of fruits, especially fruit length and shape, as well as in the total soluble solids content (°Brix). A part of strawberry, comparable results were reported for other crops, i.e., raspberry, tomato, sugar, beet, and barley [8, 32–36]. Our work, in agreement with these authors, demonstrated that PGPB treatments led to an increase equal to 21% of fruit dry weight per plant compared to the control with a significant increase in the number of fruits/cladodes. Furthermore, the bacteria inoculation significantly increased the °Brix in OFI fruits with a positive impact on fruit sweetness. The benefits of *Arthrobacter *sp. in boosting plant growth and fructification were confirmed in the second year of growth.

In general, the application of bio-stimulants may increase the synthesis of bioactive compounds in plants. Inoculation with *Arthrobacter *sp. led to a significant increase in total polyphenols, but not in flavonoid content or antioxidant activity of fruits. Similar results were shown by Esitken et al. [32] in strawberry nutritional contents and by Katsenios et al. [8] who showed an increase of total soluble solids, lycopene and total carotenoids amount which improved the tomato industrial quality. Our preliminary analysis, performed by HPLC on cactus pear fruits juices, also revealed the effect of *Arthrobacter* treatment on bioactive compound profiles. By analyzing the signal intensities detected in the samples at 280 nm, 320 nm, and 360 nm, a significant higher signal intensity was detected at 320 nm in samples from inoculated plants (see Table B_Online Resource 3). These results indicated a prevalent presence of cinnamic and hydroxycinnamic acids, chlorogenic acid, and other caffeoylquinic acids characterized by maximum absorption around this wavelength. Furthermore, fruits from plants treated with bacteria showed higher level of benzoic acids, hydroxybenzoic acids, catechins, and flavan-3-oils (maximum absorption at 280 nm) if compared to the control.

Concerning the cladodes, Ribeiro et al. [37] showed a seasonal variation on sugar amount; moreover, Da Silveira Agostini-Costa [38] reported carbohydrate, ash, and polyphenol contents were influenced by both environment and ripening stage. More recently, Messina et al. [39] found a significant and continuous increase, from March to October, of carbohydrates content in cladodes mucilage, which was negatively correlated (− 0.99, *P* < 0.01) with the water content. Our results demonstrated that samples from cladodes contained a good quantity of neutral and acid sugars in both summer and autumn seasons. In particular, samples collected in the summer presented a higher content of carbohydrates especially arabinose and mannose. It is, therefore, conceivable that the changes in the carbohydrate compositions, observed in cladodes, are related to a more efficient use of water and energy status of plants. Alterations in carbohydrate utilization and metabolism were also discussed by different authors in the case of abiotic stresses, such as nitrogen [40] or drought stress [41].

Interestingly, *Arthrobacter* enhanced the quantity of monosaccharides in cladodes, such as xylose, mannose, and arabinose when compared to samples from not inoculated plants. The latter showed a high xylose content in the autumn season, when mannose and arabinose decreased. Similar trend was observed in inoculated plants for xylose and arabinose (which were always significantly higher than control), while higher concentration of mannose was detected in treated samples in both summer and autumn. This monosaccharide, which is an epimer of the d-glucose, when associated with galactose (mannogalactans) forms the basis of complex polysaccharides, such as the mucilage with a key function in the plant hydration. These changes may be the results of the effects of PGPB, reported as enhancers of plant–water relations, plant nutritional status, and plant response to stresses, either in the rhizosphere, or as endophytes or in bio-primed plants [2, 10, 42, 43].

Nevertheless, this stimulating action, demonstrated for the first time in *Opuntia*, offers an interesting perspective to produce bio-products, such as flour with high monosaccharide contents suitable for nutraceutical purposes.

On the other hand, amounts of phenolics and flavonoids were significantly higher in OFI cladodes of not inoculated plants in both seasons. In particular, the content of flavonoids, which are the main fractions of *Opuntia* spp., showed a significant increment in summer cladodes samples of untreated plants (see also Table B_online Resources 3). Literature reports suggest that polyphenolic compounds and flavonoids act as reducing agents by being either electron donors or enzyme co-factor [44, 45]. The synthesis of these compounds can usually be enhanced in organs under stress conditions. Therefore, novel-microbe-assisted technologies can assist plants in withstanding stress conditions enhancing their tolerance and productivity. It is to note that increased temperatures decrease crop productivity by affecting biochemical, physiological, molecular, and morphological plant traits. Khan et al. [46] reported that the inoculation with the thermo-tolerant *Bacillus cereus* improved the biomass, the content, and fluorescence of chlorophylls, and antioxidant capability of soybean plants under heat stress. From this point of view, the application of PGP microbes ameliorated stress response in many crops, such as wheat, rice, maize, and rapeseed primarily by increasing nutrient availability [2, 11, 47]. In potato genotypes tolerant to heat stress, photosynthetic rates were unaffected or increased slightly at the higher temperature, while heat stress increased accumulation of foliar sucrose and decreased starch accumulation in mature leaves [48]. A similar mechanism could be hypothesized for cactus pear considering the significant accumulation of monosaccharaides in cladodes.

The application of PGPB offers an ecofriendly approach for improving crop production and counteracting the negative effects of abiotic stress [1, 47]. The soil application of *Arthrobacter* sp. on *Opuntia ficus-indica* plants improved the antioxidant activity of cladodes extracts with statistically significant differences compared to the control. The increased antioxidant activity may be attributed to the presence of antioxidant compounds, such as ascorbate (vitamin C), tocopherol (vitamin E), and flavonoids, playing important role in non-enzymatic antioxidant activity of *Opuntia* that has the capability to improve plant physiological status in response to adverse environmental factors. Further work is in progress to determine the presence of vitamins and the possible antioxidant activity by reducing groups of monosaccharides [49]

From these results, it can be reported that the application of *Arthrobacter *sp. on OFI is able to mitigate the negative effect on growth due to seasonal conditions, such as high temperatures and aridity in summer, impacting also on phenological stage, like fruit differentiation in autumn.

The results of this experiment confirmed the possibility of a wide application of *Arthrobacter* species as inoculum in different crops [2, 10, 18]. So far, this is the first report on the positive effects of this bacteria species on fruits ripening and quality of OFI.

## Conclusions

Due to its ‘multi-functionality,’ OFI is indicated as one of the species of the future, and our results may contribute to disclose its potentialities in the contest of bioeconomy and circular economy. Since natural resources and cultural practices are crucial in defining the quality of cladodes when addressed to food/nutraceutical applications, the inoculation of OFI with *Arthrobacter *sp. may be foreseen to provide a better plant growth under environmental stress conditions, or as soil fertilizer, but also to improve the synthesis of natural products, used for therapeutic applications. Further studies would be aimed to better understand the connecting pathways involved in OFI-*Arthrobacter* interaction in order to assess bacteria contribution to cactus pear metabolism and to bridge the gap in the use of *Arthrobacter *sp. from laboratory to field scale.

## Supplementary Information

Below is the link to the electronic supplementary material.

Supplementary file 1 (PDF 273 kb)

Supplementary file 2 (PDF 316 kb)

Supplementary file 3 (PDF 629 kb)

Supplementary file 4 (PDF 3063 kb)

Supplementary file 5 (PDF 3992 kb)
