# Supplementary material for: Arthrobacter sp. Inoculation Improves Cactus Pear Growth, Quality of Fruits, and Nutraceutical Properties of Cladodes
Source: Curr Microbiol. 2023 Jul 3;80(8):266. doi: 10.1007/s00284-023-03368-z (PMC10317867; doi:10.1007/s00284-023-03368-z)
Supplement: Supplementary file 1 — (PDF 273 kb) [file 284_2023_3368_MOESM1_ESM.pdf]

**TITLE:** *Arthrobacter globiformis* inoculation improves cactus pear growth, quality of fruits and nutraceutical properties of cladodes.

**JOURNAL:** *Current Microbiology*

**AUTHORS:** Platamone G., Procacci S., Maccioni O., Borromeo I., Rossi M., Bacchetta L. and Forni C.

**CORRESPONDING AUTHOR:** Loretta Bacchetta ENEA (L.B.) ENEA Casaccia, SSPT Department, BIOAG Division, Bioproducts and Bioprocesses Laboratory, Via Anguillarese 301 Rome, Italy loretta.bacchetta@enea.it

Submission ID: CMIC-D-23-00035

**SUPPLEMENTARY MATERIALS**

| Samples | New cladode area ($cm^2$) First year | New cladode area ($cm^2$) Second year |
|---|---|---|
| **Inoculated** | 82.0 | 79.0 |
| | 81.0 | 79.0 |
| | 80.0 | 81.0 |
| | 78.0 | 80.0 |
| | 80.0 | 82.0 |
| **Average cladode area (cm2)** | 80.2±1.48 | 80.5 ± 1.61 |
| **Not inoculated** | 51.0 | 51.0 |
| | 53.0 | 51,2 |
| | 52.0 | 53.0 |
| | 51.0 | 52.0 |
| | 50.0 | 52.1 |
| **Average cladode area (cm2)** | 51.4 ±1.14 | 51.8 ±0.95 |
| **Increment of cladode area in inoculated plants compared to the control (%)** | **56.03±1.31** | **57.53 ±1.28** |

**Table A Newly cladode surfaces in inoculated and not inoculated OFI plants.** The surface area of cladodes after one month from differentiation was greater in treated plants (+56.03 % ± 1.31 and +57.53 % ± 1.28 respectively in the first and the second year) when compared to the newly pad average area of the control.
