# Supplementary material for: Arthrobacter sp. Inoculation Improves Cactus Pear Growth, Quality of Fruits, and Nutraceutical Properties of Cladodes
Source: Curr Microbiol. 2023 Jul 3;80(8):266. doi: 10.1007/s00284-023-03368-z (PMC10317867; doi:10.1007/s00284-023-03368-z)
Supplement: Supplementary file 2 — (PDF 316 kb) [file 284_2023_3368_MOESM2_ESM.pdf]

TITLE: *Arthrobacter globiformis* inoculation improves cactus pear growth, quality of fruits and nutraceutical properties of cladodes.

JOURNAL: *Current Microbiology*

AUTHORS: Platamone G., Procacci S., Maccioni O., Borromeo I., Rossi M., Bacchetta L. and Forni C.

CORRESPONDING AUTHOR: Loretta Bacchetta ENEA (L.B.) ENEA Casaccia, SSPT Department, BIOAG Division, Bioproducts and Bioprocesses Laboratory, Via Anguillarese 301 Rome, Italy
loretta.bacchetta@enea.it

Submission ID: CMIC-D-23-00035

SUPPLEMENTARY MATERIALS

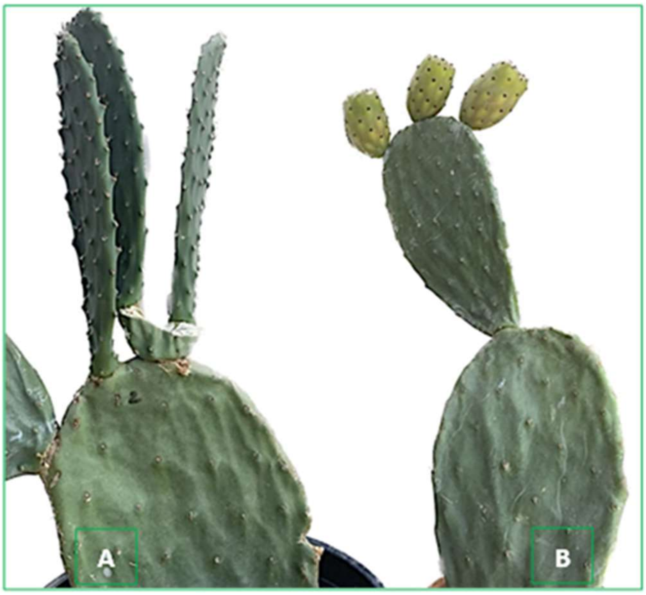

Figure A OFI plants not inoculated (A) and inoculated (B) 8 months after inoculation. Inoculated plants differentiated more cladodes of bigger sizes, and anticipated the flowering (1 - 2 months before the control), with an enhancement of the number of fruits per plants.
