# Supplementary material for: Arthrobacter sp. Inoculation Improves Cactus Pear Growth, Quality of Fruits, and Nutraceutical Properties of Cladodes
Source: Curr Microbiol. 2023 Jul 3;80(8):266. doi: 10.1007/s00284-023-03368-z (PMC10317867; doi:10.1007/s00284-023-03368-z)
Supplement: Supplementary file 3 — (PDF 629 kb) [file 284_2023_3368_MOESM3_ESM.pdf]

TITLE: *Arthrobacter globiformis* inoculation improves cactus pear growth, quality of fruits and nutraceutical properties of cladodes.

JOURNAL: *Current Microbiology*

AUTHORS: Platamone G., Procacci S., Maccioni O., Borromeo I., Rossi M., Bacchetta L. and Forni C.

CORRESPONDING AUTHOR: Loretta Bacchetta ENEA (L.B.) ENEA Casaccia, SSPT Department, BIOAG Division, Bioproducts and Bioprocesses Laboratory, Via Anguillarese 301 Rome, Italy loretta.bacchetta@enea.it

Submission ID: CMIC-D-23-00035

SUPPLEMENTARY MATERIALS

Materials and methods

The analysis of samples of cladodes and fruit from *Opuntia ficus indica* grown in pots with or without inoculation of PGPB was performed in reverse phase HPLC with an H2O/ACN gradient, acidified with 0.1% formic acid. The detection wavelengths were set at 280 nm, 320 nm and 360 nm.

Qualitative analysis of polyphenols by HPLC in cladodes and fruits

The effect of PGPB treatment on bioactive compound profile was also analyzed by HPLC which was performed on fruit and cladodes samples. During the analysis, 43 signals were detected within an elution interval of about 10-24 min. Table B reports the signal intensities detected in the samples at 280 nm, 320 nm, 360 nm.

In samples of fruits from inoculated plants, the signal intensities at 280 nm, 320 nm and 360 nm were significantly higher than those detected in samples of fruits from not treated plants. In addition to the presence of benzoic acids, hydroxybenzoic acids, catechins, flavan-3-oils and flavonoid glycosides, those results indicated the presence of different polyphenol components, such as cinnamic, hydroxycinamic acids, chlorogenic acid and other caffeoylquinic acids which have a maximum of absorption at around 320 nm wavelength.

A significant higher signal intensity was noticed at 280 nm and 360 nm in the cladode samples collected in June 2020 and 2021. These results indicate a prevalent presence of benzoic acids, hydroxybenzoic acids, catechins, flavan-3-oils and flavonoid glycosides respectively. We did not detect significant differences among the samples from inoculated and not treated plants collected during fall season.

Samples	Time of sampling	**Treatment**	Wavelengths (nm)		
			**280**	**320**	**360**
Extracts from fruits	November	not inoculated	4.084	1.731	0
		Inoculated	17.220	8.592	4.628
Extracts from cladodes	June	Not inoculated	16.437	7.286	10.884
		Inoculated	9.587	5.242	5.612
	November	Not inoculated	9.760	6.167	5.595
		Inoculated	9.259	5.272	4.971

Table B HPLC analysis: absorption intensities of fruit and cladode extracts at 280 nm, 320 nm, 360 nm. Samples collected during June and November. A significant higher signal intensity was detected at 320 nm in fruit samples from inoculated plants, while cladodes showed a significant increment in summer samples of untreated plants.
