# Supplementary material for: Arthrobacter sp. Inoculation Improves Cactus Pear Growth, Quality of Fruits, and Nutraceutical Properties of Cladodes
Source: Curr Microbiol. 2023 Jul 3;80(8):266. doi: 10.1007/s00284-023-03368-z (PMC10317867; doi:10.1007/s00284-023-03368-z)
Supplement: Supplementary file 4 — (PDF 3063 kb) [file 284_2023_3368_MOESM4_ESM.pdf]

Ann. Microbiol., **40**, 235 (1990)

Isolation and Characterization of the Bacteria Living in the Sporocarps of *Azolla filiculoides* Lam

C. FORNI*, S. GENTILI**, C. VAN HOVE***, M. GRILLI CAIOLA*

* Dipartimento di Biologia, II Università di Roma, Roma, Italia
** Dipartimento di Medicina Sperimentale, II Università di Roma, Roma, Italia
*** Lab. de Physiologie vegetale, Université Catholique de Louvain, Louvain-La-Neuve, Belgique

The presence of bacteria living together with the N_2-fixing cyanobacterium *Anabaena azollae* Strasb. in the leaf cavities of the water fern *Azolla* Lam. has been reported in many papers (1-5). The bacteria, isolated from the leaf cavities of the fern, have been identified as different species of the genus *Arthrobacter* Conn and Dimmick (6-7).

Bacteria have not been reported to be present in the sporocarps of *Azolla*; nevertheless we have recently observed by SEM that bacteria rods can be associated with *Anabaena* akinetes under the indusium of the megasporocarps (8) and of the microsporocarps of *Azolla filiculoides* Lam. (work in progress).

The present study has been undertaken to determine if the bacteria present in the sporocarps are similar to those living in the leaf cavities. For this purpose the bacteria have been isolated from the megasporocarps, microsporocarps and leaves of *A. filiculoides* Lam., and their morphological and biochemical characteristics have been determined and compared to those of the type strain *Arthrobacter globiformis* ATCC 8010.

A preliminary genus assignment of the isolated bacteria is reported.

METHODS

Sporified *Azolla filiculoides* Lam. strains ADUL FI 139, FI 162, FI 170 and FI 174 were the from *Azolla* collection, Université Catholique de Louvain, Belgium. Strain FI NA has been kindly supplied by Prof. Moretti, University of Naples, Italy.

Arthrobacter globiformis. Conn and Dimmick strain ATCC 8010 was a gift from the Pasteur Institute Culture Collection.

Isolation of bacteria. 72 megasporocarps and 23 microsporocarps were detached from *Azolla* and surface sterilized following the method of Forni *et al.* (7). The indusium caps of the sporocarps were cut whit sterile needles and plated on tryptone medium (TRN) (7).

The leaflets of *A. filiculoides* FI 139 (from the 10th to the 12th leaves) were surface sterilized and plated as previously described (7).

Petri dishes were incubated at 30 °C Bacterial colonies were detected after 4-5 days. Single colony isolates were obtained using routine bacteriological procedures.

To check the adequacy of the procedure for removing surface contaminants, both sterilized sporocarps and leaves were routinely plated on TRN medium and incubated for 5 days at 30 °C. The absence of bacterial growth after 5 days indicated a successfull surface sterilization.

Identification of bacteria and growth studies. Methods used to determine Gram classification included Gram staining and the KOH test (6) (9); *Pseudomonas stutzeri* strain OX (Prof. E. Galli, University of Milan) was used as a negative control.

To determine the nutritional requirements of the bacteria isolated, growth studies have been conducted as previously described (7). The growth factor requirements were tested by plating the bacteria on minerale medium M9 (7) supplemented with biotin or riboflavin (Merck) (10 mg 100 ml^{-1}) and casamino acids (Biolife) (1 g l^{-1}).

Enzyme assays were made as previously described (7).

Arthrobacter globiformis ATCC 8010 was used as a control throughout the study.

Identifications were based on Bergey's Manual of Systematic Bacteriology, vol. 2 (10).

Transmission Electron Microscopy (TEM). Rod stage cells were grown according to Ensign (11). Bacterial cells from culture were harvested after 4 or 8 hr. of growth in PYE medium (11), fixed, and stained with 2% phosphotungstic acid (PTA), pH 6.5, following the method of Chan *et al.* (12).

Observations were made with a Philips 300 electron microscope at 80 kV.

RESULTS

28 single colony isolates of bacteria were obtained from the sporocarps and the leaves of the five strains of *A. filiculoides*: 11 isolates from the megasporocarps, 15 from the microsporocarps and 2 from the leaves (Tab. 1).

Morphological aspects

The bacteria show a rod-coccus life cycle when grown on TRN medium, as does the type strain *A. globiformis* ATCC 8010. Spherical and rod-shaped cell

TABLE 1 — *Bacteria isolated from megasporocarps, microsporocarps and leaves of* A. filiculoides.

Azolla	Isolates
Megasporocarps	M1, M2, M3, M4, M5, M6, M7, M8, M9, M10, M11
Microsporocarps	MC1, MC2, MC3, MC4, MC5, MC6, MC7, MC8, MC9, MC10 MC11, MC12, MC13, MC14, MC15
Leaves	L1, L2

cultures also have been obtained following the methods of Ensign (11). Irregular rods which vary considerably in size and shape, including straight, bent and curved forms, are shown in Fig. 1 and 2.

As the exponential phase proceeds the rods become shorter and are replaced by cocci or coccoid cells (approximately 0.5-0.6 μm in diameter) (Fig. 1), characteristics of the stationary phase culture.

Gram-stainng is Gram-positive or Gram-negative, depending on the stage

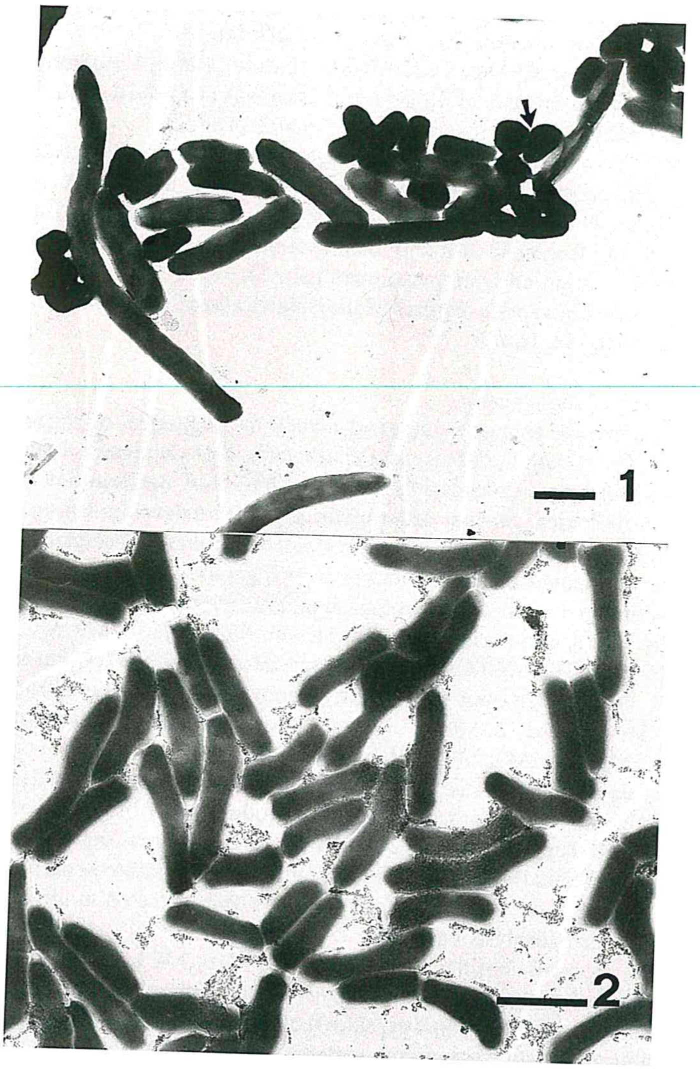

FIGS. 1-2 — Negative staining of the bacteria.
(1) Rod and coccoid cells (↑) of *A. globiformis* ATCC 8010, after 4 hr. of growth in PYE medium. Bar = 1 μm.

(2) Rod cells of the strain L1, grown for 8 hr. in PYE medium. Bar = 1 μm.

of development of each isolate. The isolates M4, M7, M10, MC11 and MC14 stain as Gram-negative, but colonies 24 hours old react as Gram-positive with the KOH test.

Enzymatic assays and growth studies of the bacteria

The bacteria produce white or bright lemon yellow colonies on nutrient media (Tab. 2).

All the cultures are catalase positive (Tab. 2). Only M6, M7, M9, M10 and MC13 isolates show a positive urease activity (Tab. 2). No arginine dehydrolase activity and acid production have been detected (Tab. 2).

The isolates M4, M8, MC7 and MC8 hydrolyze gelatin. Esculin hydrolysis was detected in ten isolates and in the type strain ATCC 8010 (Tab. 2).

Nitrate reduction was detected in 15 isolates (Tab. 2).

Growth factor is required by two isolates from megasporocarps (M3 and M10) and six isolates from microsporocarps (MC1, MC3, MC5, MC6, MC9 and MC15 (Tab. 2). The other strains are nonexacting, being able to grow on mineral medium M9 supplemented with ammonia as a nitrogen source and several carbohydrates as carbon and energy source (Tab. 2).

All the tested metabolic features of the isolates and of *A. globiformis* ATCC 8010 are reported in Tab. 3.

Classification of the bacteria

The progressive change from a rod-shaped to a spherical or coccoid form accompanied by Gram variability is a characteristic of the genus *Arthrobacter* Conn and Dimmick as described in Bergey's Manual of Bacteriology (10). The distinct morphological changes occuring during culture development in nutrient media and the biochemical characteristics place the bacteria isolated from the sporocarps and leaves of *A. filiculoides* in this genus.

Based on the described morphological and biochemical traits of the bacteria isolated, we may assign the isolates M2, M4, M5, M6, M7, M8, M9, MC2, MC4, MC10, MC11, MC13, MC14 and L1 to the species *A. globiformis* Conn and Dimmick (Tab. 4), although some isolates differ from the type strain in nitrate reduction (M2, M4, M9, MC2, MC4), in gelatin hydrolysis (M4, M8) and in esculin hydrolysis (M2, M5, M9, MC2, MC4, L1) (Tab. 2).

The species *A. nicotianae* Giovannozzi-Sermanni (isolates M1, M11, MC7, MC8, MC12 and L2) is characterized by the production of bright lemon yellow colonies on TRN medium (Tab. 2) and the ability to grow on a mineral salt medium supplemented with ammonium salt and a carbohydrate as carbon source (Tab. 2). Among these isolates differences have been detected in nitrate reduction and gelatin hydrolysis (Tab. 2).

Finally, we may consider the isolates M3, M10, MC1, MC3, MC5, MC6 and MC15 as belonging to the genus *Arthrobacter*, although these isolates cannot yet be assigned to known species, since they require casamino acids as a growth factor and produce white colonies on complex media (Tab. 2 and 3). All species listed in Bergey's Manual that require casamino acids or other growth factors form pigmented colonies on nutrient media.

The strain MC9 is also considered as *Arthrobacter* sp., because of the difficulty of identifying the species due to the lack of some of the distinguishing characteristics described in the Bergey's Manual.

TABLE 2 — *Differential characteristics and enzymatic activities of the bacteria.*

Characteristics	Isolates M1	M2	M3	M4	M5	M6	M7	M8	M9	M10	M11	MC1	MC2	MC3	MC4
Colony colour:															
white	−	+	+	+	+	+	+	+	+	+	−	+	+	+	+
lemon yellow	+	−	−	−	−	−	−	−	−	−	+	−	−	−	−
Growth factor requirement:															
casamino acids	−	−	+	−	−	−	−	−	−	+	−	+	−	+	−
Catalase	+	+	+	+	+	+	+	+	+	+	+	+	+	+	+
Urease	−	−	−	−	−	+	+	−	+	+	−	−	−	−	−
Arginine dehydrolase	−	−	−	−	−	−	−	−	−	−	−	−	−	−	−
Nitrate reduction	+	+	+	+	−	−	−	−	+	+	−	+	+	+	+
Hydrolysis of:															
esclulin	−	−	−	+	−	+	+	+	−	−	−	−	−	−	−
gelatin	−	−	−	+	−	−	−	+	−	−	−	−	−	−	−
Acid from:															
glucose	−	−	−	−	−	−	−	−	−	−	−	−	−	−	−

Characteristics	Isolates MC5	MC6	MC7	MC8	MC9	MC10	MC11	MC12	MC13	MC14	MC15	L1	L2	Type*
Colony colour:														
white	+	+	−	−	−	+	+	−	+	+	+	+	−	+
lemon yellow	−	−	+	+	+	−	−	+	−	−	−	−	+	−
Growth factor requirement:														
casamino acids	+	+	−	−	+	−	−	−	−	−	+	−	−	−
Catalase	+	+	+	+	+	+	+	+	+	+	+	+	+	+
Urease	−	−	−	−	−	W	W	−	+	W	−	−	−	−
Arginine dehydrolase	−	−	−	−	−	−	−	−	−	−	−	−	−	−
Nitrate reduction	+	+	−	−	+	−	−	−	−	−	+	−	+	−
Hydrolysis of:														
esclulin	−	−	−	−	+	+	+	−	+	+	+	−	−	+
gelatin	−	−	+	+	−	−	−	−	−	−	−	−	−	−
Acid from:														
glucose	−	−	−	−	−	−	−	−	−	−	−	−	−	−

* = *A. globiformis* ATCC 8010
W = weak

TABLE 3 — *Metabolic characteristics of the bacteria isolated and of* Arthrobacter globiformis *ATCC 8010.*

Characteristics	M1	M2	M3	M4	M5	M6	M7	M8	M9	M10	M11	MC1	MC2	MC3	MC4
	Isolates														
Utilization of:															
glucose	+	+	+	+	+	+	+	+	+	+	+	−	+	W	+
arabinose	+	−	−	+	+	+	+	−	−	−	−	−	−	−	−
mannose	+	−	−	+	+	+	+	−	−	−	−	−	−	−	−
maltose	+	−	−	−	−	+	+	−	−	−	+	−	−	−	−
mannitol	+	+	+	+	+	+	+	+	+	+	+	−	−	−	−
gluconate	+	+	+	+	+	W	−	+	+	+	−	+	+	+	+
adipate	−	+	+	+	+	−	−	−	−	−	−	+	+	+	+
malate	+	+	+	+	+	+	+	−	−	−	−	+	+	+	+
citrate	+	+	+	+	+	−	−	−	−	−	−	+	+	+	+
caprate	−	+	+	+	+	−	−	−	−	−	−	−	+	−	+
phenyl-acetate	−	+	+	+	+	+	+	+	+	+	−	+	+	+	+
N-acetyl-glucosamine	+	−	−	+	−	+	+	+	+	+	−	−	−	−	−
Characteristics	MC5	MC6	MC7	MC8	MC9	MC10	MC11	MC12	MC13	MC14	MC15	L1	L2	Type*	
	Isolates														
Utilization of:															
glucose	+	W	+	+	+	+	+	+	+	+	+	+	+	+	
arabinose	−	−	−	+	W	+	+	+	+	+	−	+	+	+	
mannose	−	−	+	+	+	+	+	+	+	+	+	+	+	+	
maltose	−	−	+	+	+	+	+	W	+	+	+	−	+	+	
mannitol	−	−	+	+	+	+	+	+	+	+	−	+	+	+	
gluconate	+	+	+	+	−	−	−	+	−	−	−	+	+	+	
adipate	+	+	+	−	−	−	−	+	−	−	−	+	−	W	
malate	+	+	+	−	+	+	+	+	+	+	+	+	+	+	
citrate	+	+	−	−	+	−	−	+	−	−	−	+	+	+	
caprate	+	−	−	−	W	−	−	−	−	−	−	+	−	−	
phenyl-acetate	+	+	+	+	−	−	−	+	−	−	−	+	+	+	
N-acetyl-glucosamine	−	−	−	+	+	+	+	+	+	+	−	−	+	+	

* = *A. globiformis* ATCC 8010
W = weak

TABLE 4 — *Distribution of* Arthrobacter *species in the sporocarps and leaves of the five strains of* A. filiculoides.

Azolla strains	*A. globiformis*	*Arthrobacter* spp.	
		A. nicotianae	*Arthrobacter* sp.
FI 162	M6 MC2 MC4	–	MC1 MC3 MC5 MC6 MC15
FI 139	M5 M9 L1	L2	MC9
FI 170	M7	M1 M11	M10
FI 174	M2 M4	–	M3
FI NA	M8 MC10 MC11 MC13 MC14	MC7 MC8 MC12	

DISCUSSION

From the megasporocarps and microsporocarps of *A. filiculoides* we have isolated bacteria like those occuring in the leaf cavities. Moreover the characteristics of these bacteria compared with those of the other bacteria living in the leaf cavities of five species of *Azolla* (5) (7) prove that these bacteria are common and constant in the association.

The isolates M5 and M9 from the megasporocarps and L1 from the leaves of *A. filiculoides* strain F1 139 are identical. We do not exclude the possibility that a bacterial selection may occur in the leaf cavities or that other bacteria may be included in the leaf cavities during its formation. The latter hypothesis may explain the presence of a bacterial population with different cell wall structures as observed by TEM (5).

On the basis of the morphological, i.e. the life cycle, the cell shape and size, the Gram staining, and biochemical characteristics of the isolated bacteria, it is likely that they belong to different species of the genus *Arthrobacter* Conn and Dimmick. But to better distinguish *Arthrobacter* from related genera, further work on peptidoglycan variation, menaquinone content and immunological analysis is in progress. Finally, we assigned 14 strains to *A. globiformis* although 8 strains are not identical to the type strain ATCC 8010, we do not consider the differences detected sufficient to assign these organisms to another species. This decision is based in part on the controversial species classification whithin this genus.

Whether *Arthrobacter* is the only bacterial genus present in the sporocarps or the physiologically most important in the symbiosis is at present unknown. However, the fact that we consistently find *Arthrobacter* in different parts of the fern suggests the possibility of a tripartite association. Moreover, the hypothesis of a coevolution of the fern, the endophyte, and the bacteria has been already put forward by Petro and Gates (13).

We thank Prof. Moretti for gift of Azolla , *Prof. E. Galli for* Ps. stutzeri *and the Pasteur Institute for providing us the* A. globiformis *ATCC 8010.*

This work was supported by CNR, special grant IPRA.

SUMMARY

Bacteria have been isolated from the megasporocarps and microsporocarps of five strains of *Azolla filiculoides*. Their morphological and biochemical characteristics have been compared to those of the bacteria living in the leaf cavities and to those of the type strain *Arthrobacter globiformis* ATCC 8010. Basing on the characteristics determined, it is likely that the isolated bacteria belong to the following species of the genus *Arthrobacter: A. globiformis* Conn and Dimmick, *A. nicotianae* Giovannozzi-Sermanni and *Arthrobacter* sp. Moreover two strains, isolated from the megasporocarps, are identical to the strain isolated from the leaf cavity.

These data suggest the constant presence of the "third component" *Arthrobacter* in the *Azolla-Anabaena* association.

RIASSUNTO

Batteri sono stati isolati dai megasporocarpi e dai microsporocarpi di cinque ceppi di *Azolla filiculoides*. Le loro caratteristiche morfologiche e fisiologiche sono state determinate e paragonate con quelle dei batteri viventi nelle cavità fogliari di *Azolla* e con quelle di *Arthrobacter globiformis* ATCC 8010.

Sulla base delle loro caratteristiche è probabile che i batteri appartengano alle seguenti specie del genere *Arthrobacter: A. globiformis* Conn and Dimmick, *A. nicotianae* Giovannozzi-Sermanni e *Arthrobacter* sp. Inoltre due ceppi, isolati dai megasporocarpi, sono identici al ceppo isolato dalle cavità fogliari della stessa pianta. I dati suggeriscono la costante presenza del "terzo componente" *Arthrobacter* nell'associazione *Azolla-Anabaena*.

REFERENCES

(1) Grilli M.: *Infrastrutture di* Anabaena azollae *vivente nelle foglioline di* Azolla caroliniana. Ann. Microbiol., **14**, 69 (1964).

(2) Peters G.A., Toia R.E. jr., Raveed D., Levine N.J.: *The* Azolla-Anabaena azollae *relationship. VI Morphological aspects of the association.* New Phytol., **80**, 583 (1978).

(3) Gates J.E., Fisher R.W., Candler R.A.: *The occurrence of coryneform bacteria in the leaf cavity of* Azolla. Arch. Microbiol., **127**, 163 (1980).

(4) Grilli Caiola M., Albertano P.: *Recognition mechanisms in the* Azolla-Anabaena *symbiosis,* in "Cell to cell signals in Plant, Animal and Microbial Symbiosis" (S. Scannerini, D. Smith, P. Bonfante-Fasolo, V. Gianinazzi-Pearson eds.), p. 27, Springer Verlag (1988).

(5) Grilli Caiola M., Forni C., Castagnola M.: *Bacteria in the* Azolla-Anabaena azollae *association.* Symbiosis, **5**, 185 (1988)

(6) Wallace W.H., Gates J.E.: *Identification of eubacteria isolated from the leaf cavities of four species of the N-fixing* Azolla *fern as* Arthrobacter *Conn and Dimmick.* Appl. Environ. Microbiol., **52**, 425 (1986).

(7) Forni C., Grilli Caiola M., Gentili S.: *Bacteria in the* Azolla-Anabaena *symbiosis,* in "Nitrogen fixation with non-legumes" (F.A. Skinner, R.M. Boddey, I. Fendrik, eds.), p. 83, Kluwer Academic Publisher (1989).

(8) Forni C., Grilli Caiola M., Gentili S.: *Bacteria in the megasporocarps of* Azolla filiculoides *Lam.* Giorn. Bot. Ital., **124**, (in press).

(9) Gregersen T.: *Rapid method for the distinction of the Gram-negative from the Gram-positive bacteria.* Eur. J. Appl. Microbiol. Biotechnol., **5**, 123 (1978).

(10) Keddie R.M., Collins M.D., Jones D.: *Genus* Arthrobacter *Conn and Dimmick,* in "Bergey's Manual of Systematic bacteriology", vol. 2 (P.H.A. Sneath, N.S. Mair, M.E. Sharpe, J.G. Holt eds.), p. 128, Williams and Wilkins, Baltimore (1966).

(11) Ensign J.: *Long-term starvation survival of rod and spherical cells of* Arthrobacter crystallopoietes. J. Bacteriol., **103**, 569 (1970).

(12) Chan E.C.S., Gomersall M., Bernier J.: *The negative staining of "difficult" bacteria like* Arthrobacter globiformis *for electron microscopy.* Can. J. Microbiol., **20**, 901 (1974).

(13) Petro M.G., Gates J.E.: *Distribution of* Arthrobacter *sp. in the leaf cavities of four species of the N-fixing* Azolla *fern.* Symbiosis, **3**, 41 (1987).

(Pervenuto in Redazione il 28 giugno 1990).
