# Supplementary material for: Arthrobacter sp. Inoculation Improves Cactus Pear Growth, Quality of Fruits, and Nutraceutical Properties of Cladodes
Source: Curr Microbiol. 2023 Jul 3;80(8):266. doi: 10.1007/s00284-023-03368-z (PMC10317867; doi:10.1007/s00284-023-03368-z)
Supplement: Supplementary file 5 — (PDF 3992 kb) [file 284_2023_3368_MOESM5_ESM.pdf]

MICROBIOLOGICA, 15, 271-280, 1992

THE MORPHOGENESIS OF *ARTHROBACTER* SPECIES ISOLATED FROM THE FERN *AZOLLA FILICULOIDES* LAM

FORNI, C., and GRILLI CAIOLA, M.

Dipartimento di Biologia, Università di Roma Tor Vergata, Via E. Carnevale, 00173 Roma; Italy

Received December 31, 1991 *Accepted* February 15, 1992

SUMMARY

The morphogenesis and life cycle of *Arthrobacter* sp. strains AF1 and AF2, isolated from the leaf cavities of *Azolla filiculoides* Lam, have been studied by light and electron microscopy. The strains are morphologically similar to the type strain *Arthrobacter globiformis* ATCC 8010. They are straight or bent rods in the log phase and cocci in the stationary growth stage. The cocci derive from the fragmentation of the rod inside an electron transparent mother cell wall. Thin sections of the bacteria show an electron dense cell wall overlying the plasma membrane. Evidence for trilaminar region characteristics of most gram-negative bacterial cell walls, was totally lacking in the three strains.

KEY WORDS Arthrobacter, *morphogenesis,* Azolla filiculoides

INTRODUCTION

In the leaf cavities of the fern *Azolla*, the symbiont *Anabaena* lives together with a bacterial population (Grilli, 1964; Gates *et al.,* 1980; Grilli Caiola *et al.,* 1988). These eubacteria have been reported as belonging to the following different genera: *Alcaligenes, Caulobacter* (Newton and Herman, 1979), *Arthrobacter* (Wallace and Gates, 1986; Forni *et al.,* 1989, 1990) and *Agrobacterium* sp. (Plazinski *et al.,* 1990). Moreover, bacteria classified as *Arthrobacter* have been isolated from the leaf cavities of different species of

Azolla (Wallace and Gates, 1986; Petro and Gates, 1987; Forni *et al.,* 1989) as well as from the sporocarps of *A. filiculoides* Lam (Forni *et al.,* 1990). The genus *Arthrobacter* Conn and Dimmick (1947) includes bacteria characterized by a rod-coccus cycle. The rods appear during the log phase of growth when the growth rate is maximum (Luscombe and Gray, 1971), while the coccoid cells are typical of the lag and stationary phase. This genus is also notorious for the Gram variability that results in a number of Gram-negative cells in a Gram-positive culture. This Gram variability is shown even under optimal growth conditions (Beveridge, 1990). Reports in the literature on the morphological and ultrastructural characterization of the bacteria living in *Azolla* are few and contradictory. Grilli Caiola *et al.* (1988) reported the presence in the leaf cavities of *A. caroliniana* Willd of rod and coccus shaped bacteria. The cell wall had a peptidoglycan layer, different in thickness but no Gram-negative bacteria were detected. On the contrary Nierzwicki-Bauer *et al.* (1990) reported the presence of different shaped Gram-negative bacteria in *A. mexicana* Presl. and *A. mexicana* - *Anabaena* free.

The present study had two objectives. The first was to study the life cycle in culture of the bacteria strains, isolated from the leaf cavities of *Azolla filiculoides* Lam. The second was to obtain an ultrastructural characterization of the strains during morphogenesis in order to have a better knowledge of these bacteria isolated from *Azolla*. Moreover, since the isolated bacteria have been classified as *Arthrobacter* sp. Conn and Dimmick the results obtained are compared with an examination of the type species *Arthrobacter globiformis* ATCC 8010.

MATERIALS AND METHODS

Bacteria strains

Bacterial strains AF1 and AF2 were isolated from the leaf cavities of *A. filiculoides* Lam. The isolation of the bacteria was performed following the methods previously described (Forni *et al.,* 1989). Enzyme assays and growth studies were conducted as previously described (Forni *et al.,* 1989). The strains were characterized and classified, according to the Bergey's Manual of Bacteriology (1986), as *Arthrobacter* sp. Conn and Dimmick.

The type strain *Arthrobacter globiformis* ATCC 8010 was kindly provided by the Pasteur Institute of Paris.

Life cycle

Coccoid cells in stationary phase were inoculated into TYE medium (tryptone 10 g/l; yeast extract 1 g/l, pH 7) and incubated in a shaker at 150 rpm (New Brunswick Orbital shaker) at 30°C. The growth of each culture was followed by optical density at 660 nm so that samples could be taken at selected growth phases for Gram staining and processing for electron microscopy. The KOH test was also used for Gram classification (Wallace and Gates, 1986).

Scanning Electron Microscopy (SEM)

Samples of cultures in different growth phases were fixed in 3% glutaraldehyde in 0.1M sodium cacodilate buffer, pH 6.9 at 4°C for 24 hrs. The samples were postfixed in 1% OsO_4 in 0.1M sodium cacodylate buffer for 2 hrs, dehydrated in a graded series of acetone, critical point dried and covered with colloidal gold. The observations were made by a Scannosan Electron Microscope.

Transmission Electron Microscopy (TEM)

The bacterial cells in log and stationary growth phase were harvested by centrifugation (1600 g for 10 min), and fixed in 2.5% glutaraldehyde in 0.1M phosphate buffer, pH 7, for 5 hrs. The samples were postfixed overnight in 1% OsO_4 in 0.1M phosphate buffer at 4°C. The postfixed samples were dehydrated in different percentages of ethanol and embedded with EPON 812 (Agar 100 Resin). Ultrathin sections were stained with 1% uranyl acetate for 15 min and with lead citrate (Reynolds, 1963). The presence of glycogen granules was determined by Thiéry's reaction (Thiéry, 1967).

For the negative staining one drop of the cell suspension, obtained as described by Chan *et al.* (1974), was stained with 2% PTA, pH 6.5 for 10 min. The grids were washed with sterile double distilled water and air dried.

The observations were made under a Philips 400 electron microscope.

RESULTS

Life cycle and morphogenesis

The growth curves of the strains AF1, AF2

and ATCC 8010 grown in TYE medium are shown in Fig. 1. These strains showed a lag phase of 2-3 hrs and a log growth phase of 11-14 hrs, followed by a stationary phase (Fig. 1). The average doubling times were the following: 1.7 hrs for AF1, 2 hrs for AF2 and 1.8 hrs for ATCC 8010.

On inoculation, the strains AF1, AF2 and ATCC 8010 were cocci Gram+ of 0.6-1.2 μm in diameter (Fig. 2 a, a'); during the first two hours after the inoculation, there was no significant change in cell shape. At the third hour the cocci began to elongate to form short rods. The Gram positive and sometimes Gram variable rods increased in length during the log phase (Fig. 2 b, b'). During this phase "V" and "Y" forms were observed. At the end of the log phase, the endogenous cell division and fragmentation of the rods produced coccoid cells typical of the stationary phase (Fig. 2 c, c').

Ultrastructural characteristics of Arthrobacter *during morphogenesis*

SEM observations

The pleomorphism of the bacteria detected in the light microscope was confirmed by the SEM observations. In fact strains AF1 and AF2 appeared as rods or coccoid cells with a smooth surface and lacking in flagella (Fig. 3, a). In the transition phase, constrictions were visible along the rods. These rings were probably due to the fragmentation of the rod into several coccoid cells.

Sometimes the cocci were more like coccoid cells (Fig. 3b). Both morphological forms of the two strains were similar to those of the type strain ATCC 8010 (Fig. 3b), which showed straight or bent rods, sometimes very long, and cocci. Budding was also observed.

TEM observations

In the log phase of growth, the cells of AF1 and AF2 stained with PTA appeared as rods (Fig. 4a) similar to those of the type strain (Fig. 4b). The length of the rods ranged between 1.8 - 3.4 μm and sometimes longer, while the diameter was within the range 0.4-0.6 μm. No pili, fimbriae or flagella were detectable.

The long rods contained electron-transparent areas alternating with electron-dense ones due to the formation of cocci.

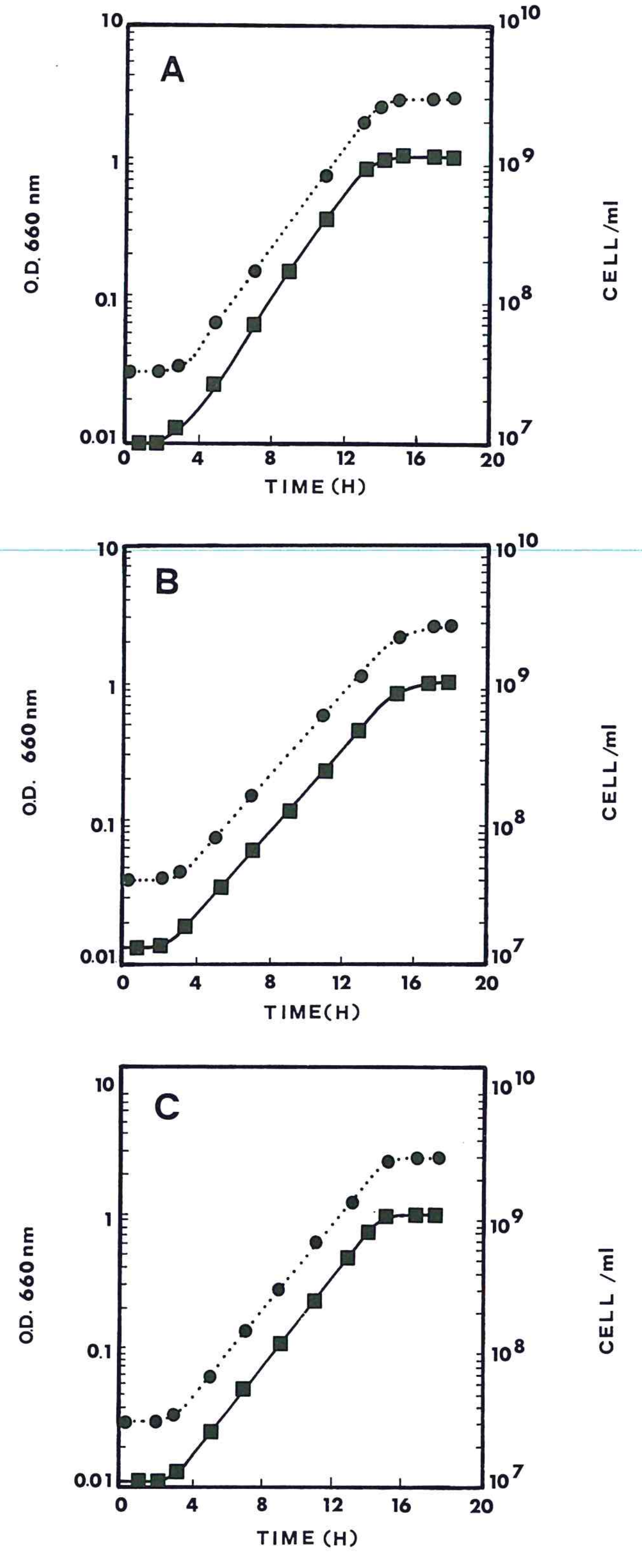

FIGURE 1 - Representative growth curves of *Arthrobacter* strains: AF1 (a), AF2 (b) ATCC 8010 (c) in TYE medium. ■——■ Cell/ml; ●- - -● OD 660 nm.

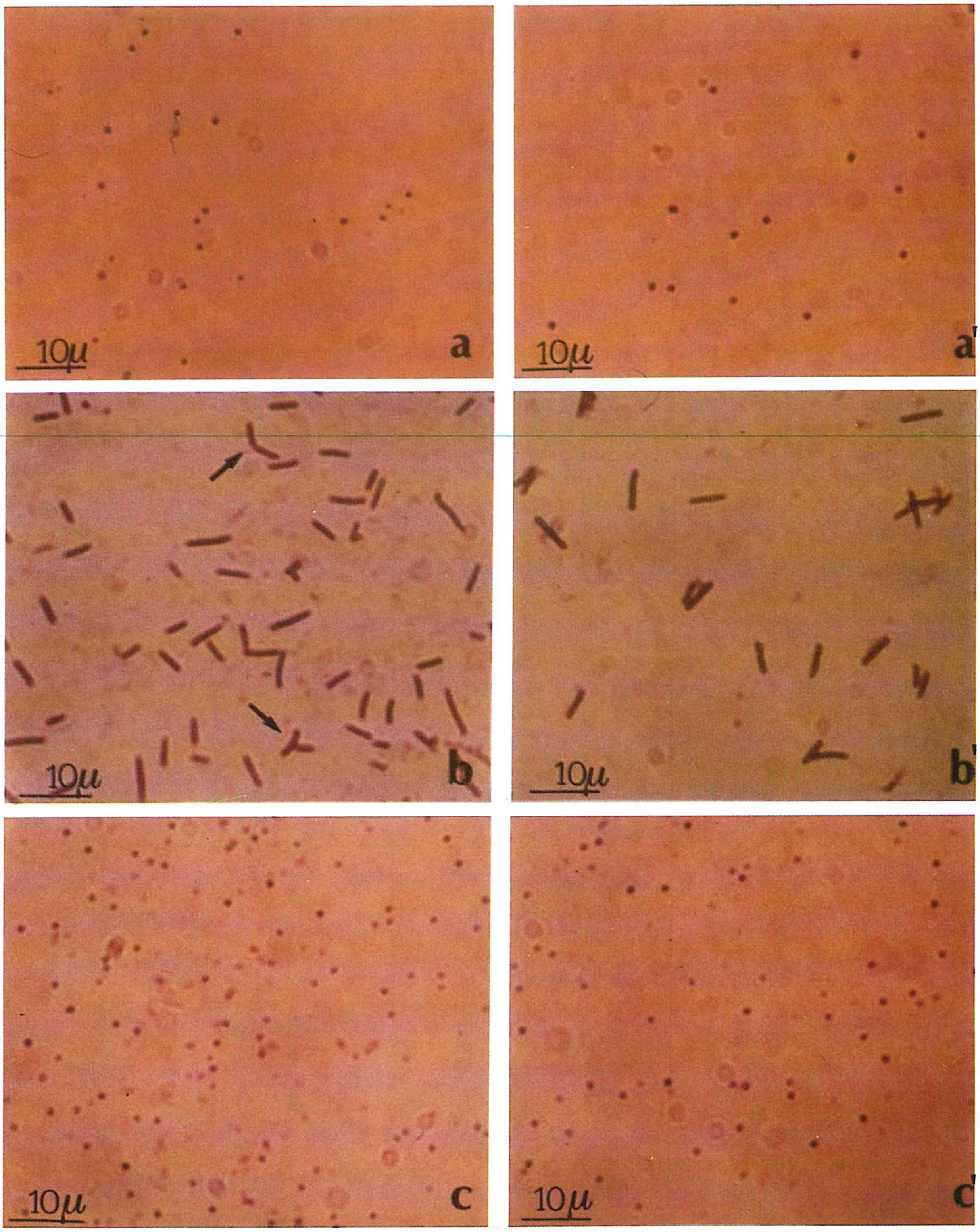

FIGURE 2 - Morphogenetic cycle of AF1 (a,b,c) and ATCC 8010 (a',b'c')
(a,a'): cocci during the lag phase; (b,b'): rods during the log phase. V and Y forms can be seen in b (→); (c,c'): cocci produced at the end of growth cycle.

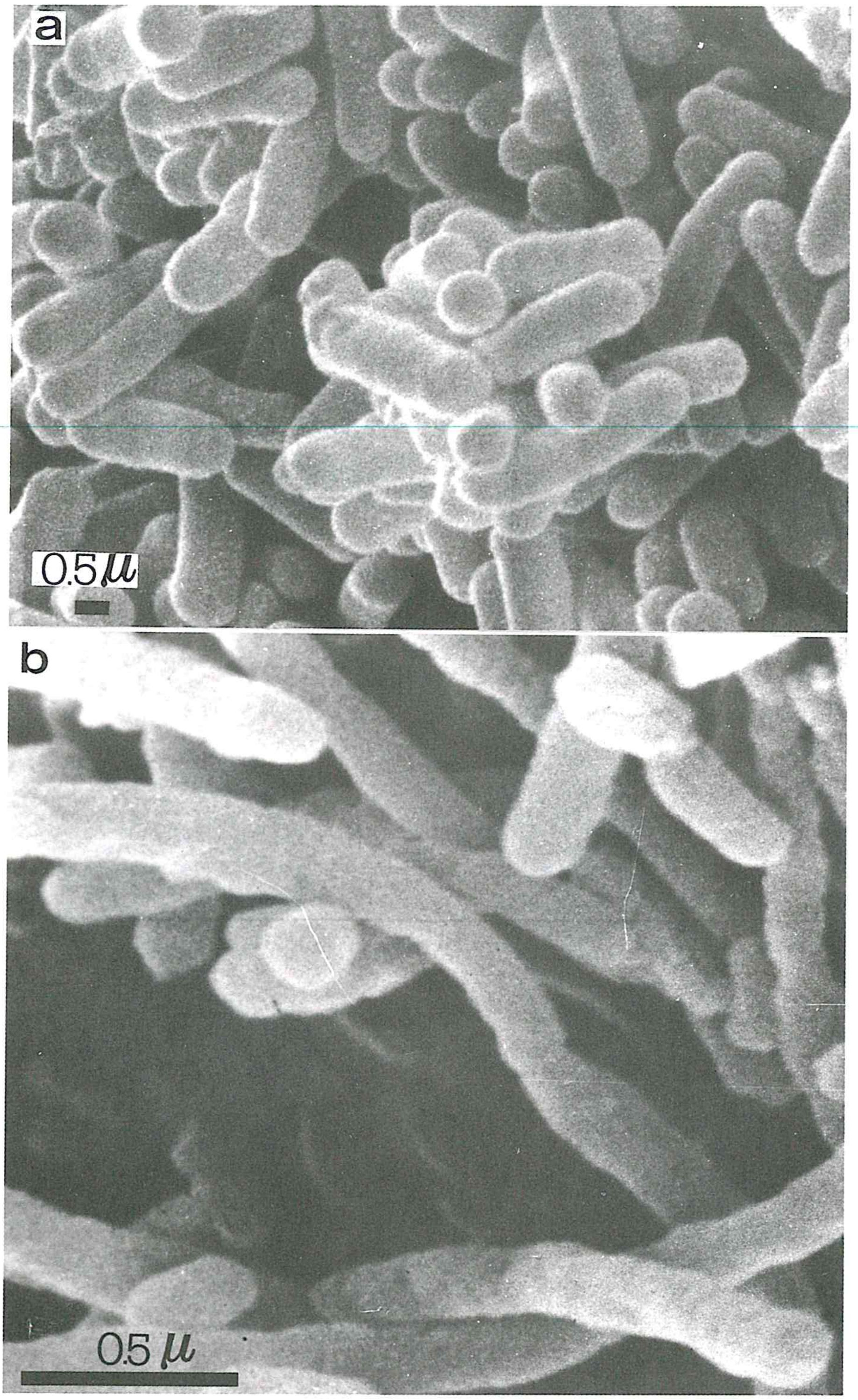

FIGURE 3 - SEM micrographs of the cultures of AF2 (a) and ATCC 8010 (b)

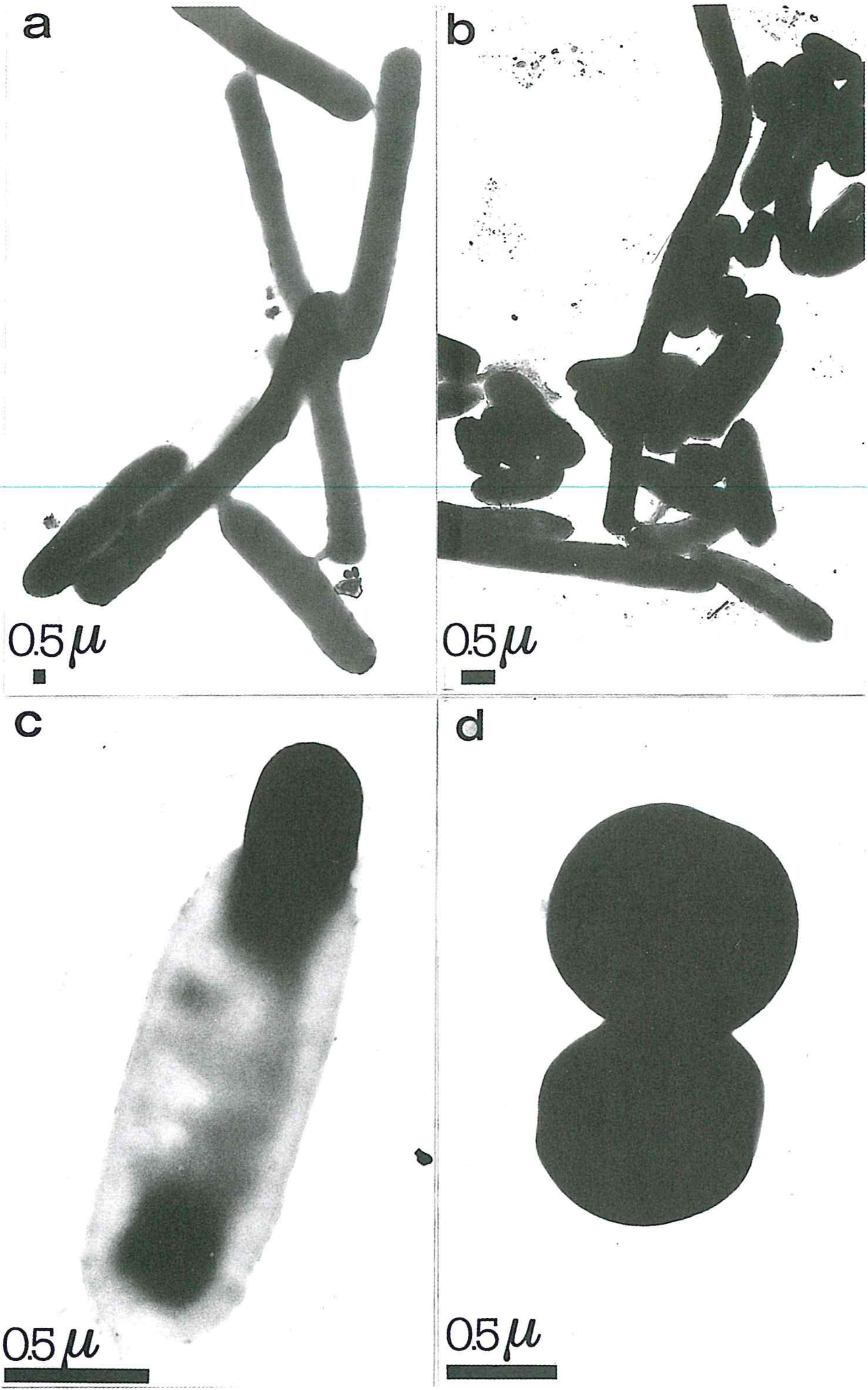

FIGURE 4 - TEM micrographs of negative stained bacteria:
a) rods of AF1; b) rods of ATCC 8010; c) Coccus formation of AF2 within an electron transparent mother cell; d) Coccoid cells of ATCC 8010.

In fact, at the end of the log phase, coccoid cells were originated by division of the rod cell mother cytoplasm (Fig. 4c). Enlarged coccoid cells were present (Fig. 4d).
In thin sections the thickness of the cell wall in both cell types varied (Fig. 5); sometimes a slime production outside of the cells was visible. The outer membrane was not observed.
The rod cytoplasm of AF1 (Fig. 5a) and ATCC 8010 (Fig. 5b) contained glycogen granules and areas of fibrillar material corresponding to the nucleoplasm. Polyphosphate granules were not detectable.

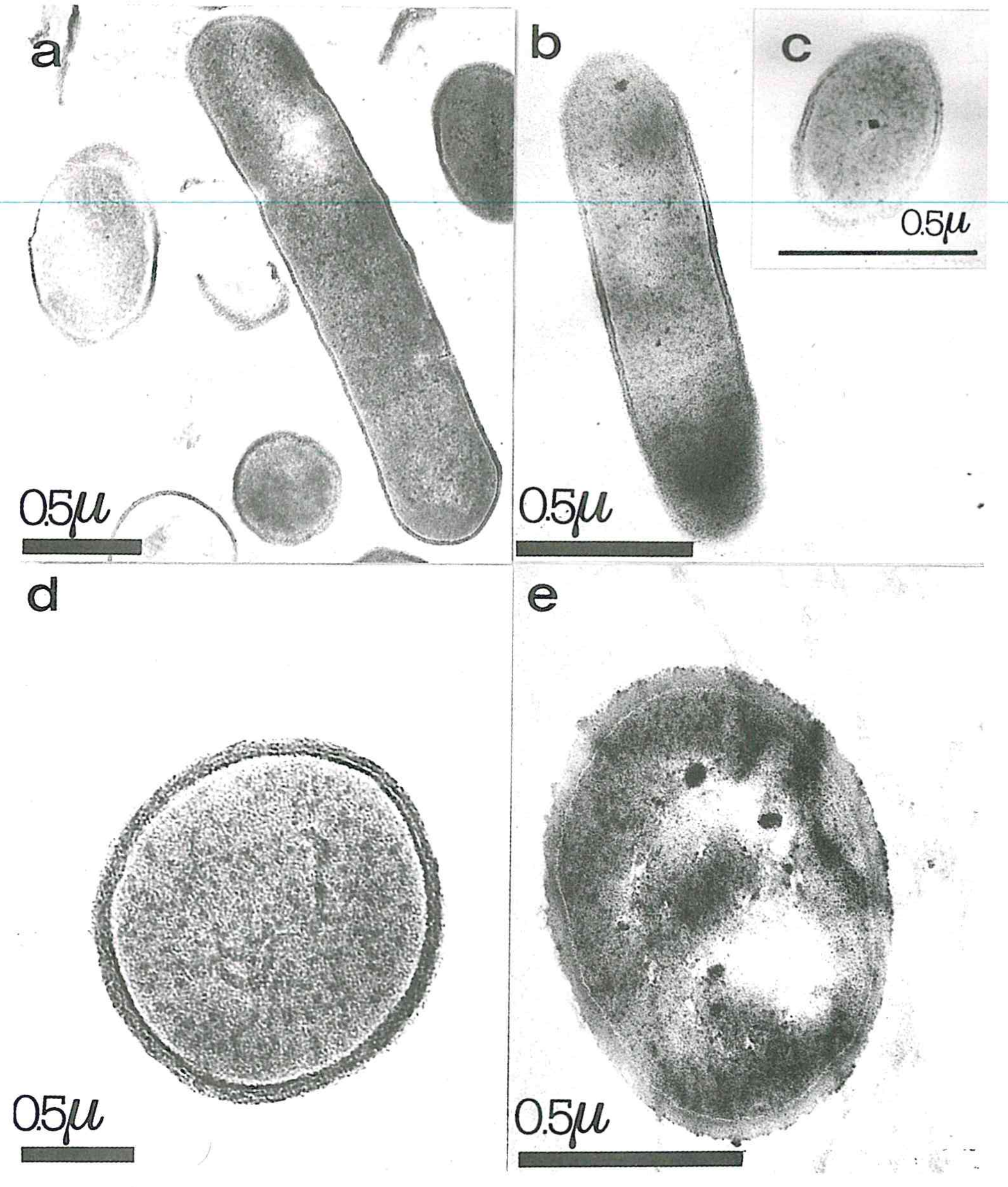

FIGURE 5 - TEM micrographs of ultrathin sections:
a) rods of AF1 in longitudinal and transverse thin section. The cell wall has a thin peptidoglycan layer; b) longitudinal and c) transverse thin section of rods of ATCC 8010; d) Coccus of AF1 with an evident thick cell wall as in the Gram-positive bacteria; e) Coccus of ATCC 8010.

DISCUSSION

The Gram staining and morphology of the strains AF1 and AF2, isolated from the leaf cavities of *A. filiculoides*, are similar to the type strain of the genus *Arthrobacter*. In addition, cellular differentiation as exhibited by members of this genus (Ward and Claus, 1973; Lucas and Clark, 1975; Duxbury *et al.,* 1977), was also determined in the strains AF1 and AF2. In TYE medium the growth cycle of the three strains is completed in a day.

In these microorganisms the sphere-rod morphogenesis involves changes in the length of the cells other than in the ultrastructure of the cell wall. In fact thin sections of both morphological forms reveal an electron-dense homogeneous structure of varying thickness, which immediately overlays the plasma membrane. The trilaminar portion of the Gram-negative bacteria was not observed.

Differences in the cell wall thickness in *Arthrobacter* have been already reported by Ward and Claus (1973) and according to these authors may make an important contribution to the Gram-staining variability typical of this genus and also determined in the strains AF1 and AF2. Nevertheless it has to be ascertained whether there is any biochemical variation in the composition of the cell wall of the two morphological forms. Krulwich *et al.* (1967a,b) reported that in *A. crystallopoietes* the rod-coccus morphogenesis involved changes in the length of polysaccharides and differences in the cross-bridges of the glycopeptides. Immunological studies may add further contributions to these data. Preliminary data have shown similar antigenic reactions in both rod and coccus forms (work in progress).

The pleomorphism and morphogenesis of these bacteria in culture might explain the remarkable differences observed in the ultrastructure of the bacteria in the symbiosis (Grilli Caiola *et al.,* 1988). In fact SEM and TEM observations of the leaf cavities of *A. caroliniana* have shown the presence of rod and coccus shaped bacteria. In addition, the bacterial cell wall had a peptidoglycan layer of different thickness.

Literature reports on the formation of the coccus described the progressive divisions of the rod resulting in a gradual decrease in cell size until at the end of the cycle cocci are produced (Stevenson, 1961). The data of this study indicate that the cocci derive from an internal division of the rod mother cell cytoplasm. It is likely that this phase is only present for a relatively short period, making studies on this stage very difficult and this paper is the first proof of the presence of such a structure in *Arthrobacter*.

REFERENCES

BERGEY'S. (1986). Manual of Systematic Bacteriology, vol. 2, 9th ed., Williams and Wilkins, Baltimore.

BEVERIDGE, T.J. (1990). Mechanism of Gram variability in select bacteria. *Journal of Bacteriology* **172,** 1609-1620.

CHAN, E.C.S., GOMERSALL, M., and BERNIER, J. (1974). The negative staining of "difficult" bacteria like *Arthrobacter globiformis* for electron microscopy. *Canadian Journal of Microbiology* **20,** 901-903.

CONN, H.J., and DIMMICK, I. (1947). Soil bacteria similar in morphology to *Mycobacterium* and *Corynebacterium. Journal of Bacteriology* **54,** 291-303.

DUXBURY, T., GRAY, T.R.G., and SHARPLES, G.P. (1977). Structure and chemistry of walls of rods, cocci and cystites of *Arthrobacter globiformis. Journal of General Microbiology* **103,** 91-99.

ENSIGN, J.C., and WOLFE, R.S. (1964). Nutritional control of morphogenesis in *Arthrobacter crystallopoietes. Journal of Bacteriology* **87,** 924-932.

FORNI, C., GRILLI CAIOLA, M., and GENTILI, S. (1989). Bacteria in the *Azolla-Anabaena* symbiosis. In: "Nitrogen fixation with non-legumes". Skinner, F.A., *et al.,* Eds. Kluwer Academic Publisher, Dordrecht, The Netherlands, pp. 83-88.

FORNI, C., GENTILI, S., VAN HOVE, C., and GRILLI CAIOLA, M. (1990). Isolation and characterization of the bacteria living in the sporocarps of *Azolla filiculoides* Lam. *Annali Microbiologia Enzimologia* **40,** 235-243.

GRILLI, M. (1964). Infrastrutture di *Anabaena azollae* vivente nelle foglioline di *Azolla caroliniana. Annali Microbiologia Enzimologia* **14,** 69-90.

GRILLI CAIOLA, M., FORNI, C., and CASTAGNOLA, M. (1988). Bacteria in the *Azolla-Anabaena* association. *Symbiosis* **5,** 185-198.

KOLENBRANDER, P.E., and HOHMAN, R.J. (1977). Electron microscopic study of cell surface rings during cell division and morphogenesis of *Arthrobacter crystallopoietes*. *Journal of Bacteriology* **130**, 1345-1356.

KRULWICH, T.A., ENSIGN, J.C. TIPPER, D.J., and STROMINGER, J.L. (1967a). Sphere-rod morphogenesis in *Arthrobacter crystallopoietes*. I. Cell wall composition and polysaccharides of the peptidoglycan. *Journal of Bacteriology* **94**, 734-740.

KRULWICH, T.A., ENSIGN, J.C., TIPPER, D.J., and STROMINGER, J.L. (1967b). Sphere-rod morphogenesis in *Arthrobacter crystallopoietes* II. Peptides of the cell wall peptidoglycan. *Journal of Bacteriology* **94**, 741-750.

LUCAS, D.S., and CLARK, J.B. (1975). Induction of morphogenesis in the genus *Arthrobacter*. *Journal of Bacteriology* **124**, 1034-1036.

LUSCOMBE, B.M., and GRAY, T.R.G. (1971). Effect of varying growth rate on the morphology of *Arthrobacter*. *Journal of General Microbiology* **69**, 433-434.

NEWTON, J.W., and HERMAN, H.I. (1979). Isolation of cyanobacteria from the aquatic fern *Azolla*. *Archives of Microbiology* **120**, 161-165.

NIERZWICKI-BAUER, S., and AULFINGER, H. (1990). Ultrastructural characterization of eubacteria residing within leaf cavities of symbiotic and cyanobiont-free *Azolla mexicana*. *Current Microbiology* **21**, 123-129.

PETRO, M.J., and GATES, J.E. (1987). Distribution of *Arthrobacter* sp. in the leaf cavities of four species of the N-fixing *Azolla* fern. *Symbiosis* **3**, 41-48.

PLAZINSKI, J., TAYLOR, R., SHAW, W., CROFT, L., ROLFE, B.G., and GUNNING, B.E.S. (1990). Isolation of *Agrobacterium* sp. strain from the *Azolla* leaf cavities. *FEMS Microbiology Letters* **70**, 55-60.

REYNOLDS, E.S. (1963). The use of lead citrate at high pH as an electron opaque stain in electron microscopy. *Journal of Cell Biology* **17**, 208-213.

STEVENSON, I.L. (1961). Growth studies on *Arthrobacter globiformis*. *Canadian Journal of Microbiology* **7**, 569-575.

THIÉRY, J.P. (1967). Mise en evidence des polysaccharides sur coupe fines en microscopie électronique. *Journal de Microscopie* **6**, 987-1018.

WALLACE, W.H., and GATES, J.E. (1986). Identification of eubacteria isolated from the leaf cavities of four species of the N-fixing *Azolla* fern as *Arthrobacter* Conn and Dimmick. *Applied Environmental Microbiology* **52**, 425-429.

WARD, C.M., and CLAUS, G.W. (1973). Gram characteristics and wall ultrastructure of *Arthrobacter crystallopoietes* during coccus-rod morphogenesis. *Journal of Bacteriology* **114**, 378-389.

L. 8.235
(Iva assolta dall'Editore
ai sensi dell'Art. 74 lettera C del DPR 633/72)
